# Abnormality of the DNA double-strand-break checkpoint/repair genes, *ATM, BRCA1* and *TP53*, in breast cancer is related to tumour grade

**DOI:** 10.1038/sj.bjc.6601804

**Published:** 2004-04-20

**Authors:** S L Ding, L F Sheu, J C Yu, T L Yang, B F Chen, F J Leu, C Y Shen

**Affiliations:** 1 Graduate Institute of Life Sciences, National Defense Medical Center, Taipei 114, Taiwan; 2 Institute of Biomedical Sciences, Academia Sinica, Taipei 115, Taiwan; 3 Department of Pathology, Tri-Service General Hospital, National Defense Medical Center, Taipei 114, Taiwan; 4 Department of Surgery, Tri-Service General Hospital, National Defense Medical Center, Taipei 114, Taiwan; 5 Department of Surgery, Mackay Memorial Hospital, Taipei 104, Taiwan; 6 Department of Pathology, Mackay Memorial Hospital, Taipei 104, Taiwan; 7 Section of Pathology, Cardinal Tien Hospital and Fu-Jen Catholic University, Taipei 231, Taiwan

**Keywords:** *ATM*, *BRCA1*, *TP53*, breast cancer

## Abstract

The role of the DNA double-strand-break (DSB) checkpoint/repair genes, *ATM*, *BRCA1* and *TP53*, in sporadic breast cancer requires clarification, since *ATM* and *BRCA1* mutations are rare in sporadic tumours. In an attempt to explain this phenomenon, we postulated that (i) in addition to genetic deletion, abnormal expression of DSB checkpoint/repair proteins might abolish the function of these genes and (ii) there might be a combined effect of individual defective genes during breast cancer pathogenesis. Using a largely homogenous group of 74 specimens of early-onset (**⩽**35 years of age) infiltrating ductal carcinomas, we examined associations between pathological grade and genetic deletion and/or abnormal protein expression of *ATM, BRCA1* and *TP53*. The results showed that high-grade tumours displayed a high frequency of loss of heterozygosity (LOH) at, and/or abnormal expression of, *ATM*, *BRCA1* and *TP53*. Multigenetic analysis showed abnormalities in *BRCA1* to be independently associated with high-grade tumours. *ATM* and *TP53* appeared to play an assistant role, abnormalities in these genes significantly increasing the possibility of poor differentiation in tumours with abnormalities in *BRCA1*. Furthermore, a higher number of abnormalities (LOH or abnormal expression) in these three genes correlated with poor tumour differentiation. Thus, this study suggests that combined changes in several DSB checkpoint/repair genes belonging to a common functional pathway are associated with breast cancer pathogenesis.

Tumorigenesis results from a series of genomic alterations that leads to progressive disorder of the normal mechanisms controlling cell growth, death and differentiation (Bishop, 1991). Our recent studies have demonstrated that the extent of DNA double-strand-break (DSB)-initiated genomic deletion in tumours is significantly increased in high-grade breast tumours (Shen *et al*, 2000). On the basis of these findings, we hypothesised that *ATM, BRCA1* and *TP53*, the critical genes in the DSB checkpoint/repair pathway, might play an important role during breast tumorigenesis, and that defects in these genes could result in poor tumour differentiation. A causal link between breast cancer development and mutation of *ATM, BRCA1* and *TP53* has been found in familial breast cancer syndromes (Buchholz *et al*, 1999). However, the probability of finding *ATM* and *BRCA1* mutations in sporadic breast cancer is low (Vorechovsky *et al*, 1996; Bay *et al*, 1998; Papa *et al*, 1998). In contrast, genomic deletions at the loci harbouring these three genes are relatively frequent (Lo *et al*, 1998; Rio *et al*, 1998; Shen *et al*, 2000). Genomic deletion represents one of the ‘two hits’ needed to inactivate tumour suppressor genes for cancer formation (Knudson, 1971). Recent studies have suggested that epigenetic mechanisms, manifested as abnormal protein expression, serve as the other hit, and are involved in abrogating the function of certain tumour suppressor genes (Jones and Baylin, 2002). In addition, as our understanding of cancer development extends beyond single-gene disorders to multigenetic disorders and aetiological pathway-wide abnormalities, it is tempting to speculate that the combined effect of several defective genes in a common antitumour pathway could lead to more poorly differentiated tumours. We therefore carried out the present study, using a highly homogenous study population and a multigenetic design, to examine the relationship between abnormalities (both genetic deletion and abnormal expression) in the most critical DSB checkpoint (*ATM* and *TP53*) and repair (*BRCA1*) genes and tumour grade.

## MATERIALS AND METHODS

### Patients and specimens

The study subjects, 74 Chinese women with early-onset (**⩽**35 years of age) breast cancer, were a subset of patients selected from our ongoing hospital-based breast cancer cohort (Yang *et al*, 1997; Shen *et al*, 2000). Breast cancer in Taiwanese (Chinese) women is characterised by a low incidence (Yang *et al*, 1997), early tumour onset (Lo *et al*, 1998) and novel genomic alterations (Lou *et al*, 1997; Shen *et al*, 2000). Owing to the low incidence of breast cancer, which suggests an overall lower effect of common risk factors, and its homogenous genetic background, the Taiwanese population has certain advantages for studying the effects of genetic variations. In order to obtain an aetiologically homogenous group of study subjects, which should help in elucidating the causes of tumour, we restricted our study to histologically confirmed, primary infiltrating ductal carcinomas (IDCs). Direct sequencing showed that none of the patients had germline mutation of the *BRCA1* gene. Furthermore, no patient had a family history of breast cancer or of Li–Fraumeni syndrome (hereditary, and associated with mutant *TP53*) in their first-degree relatives, and no case of germline mutations in the *ATM* gene has ever been identified in breast cancer families in Taiwan, so these tumours were most likely sporadic. On grading using the Scarff–Bloom–Richardson system (Le Doussal *et al*, 1989), the number of IDCs of each grade was found to be similar. Institutional review board-approved informed consent was obtained from each patient prior to tissue collection. None of the patients had received neoadjuvant treatment or preoperative chemotherapy or radiotherapy, which could have caused up- or downregulation of gene expression. Paraffin-embedded tumour tissue and peripheral blood were obtained from each patient. To ensure that the tumour tissue samples assayed consisted of more than 90% tumour cells, laser capture microdissection using a PixCell laser capture microscope (Arcturus Engineering, Mountain View, CA, USA) was routinely performed on slides to collect the scattered tumour cells without normal cells. Somatic DNA was extracted from tumour tissue and genomic DNA from peripheral blood (normal control) using a conventional proteinase K-phenol/chloroform protocol (Shen *et al*, 2000). All specimens were stored at −80°C until analysed.

### Allelotyping PCR and definition of loss of heterozygosity (LOH)

**Figure 1:**
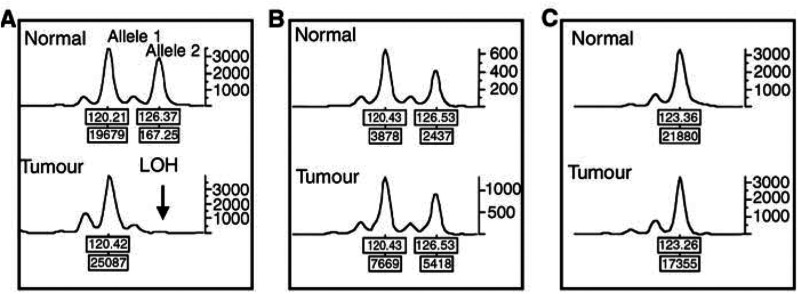
Allelotyping PCR to detect LOH of *BRCA1* (trinucleotide marker D17S1322) in three representative breast tumours. The locus of the marker was considered to show LOH when a four-fold or greater difference was seen in the relative allele intensity ratio (allele 1 : allele 2) between the tumour and normal DNA. Tumour (**A**) showed LOH at this locus. Tumour (**B**) showed a relative allele intensity ratio for tumour and normal DNA of 1.12, and was considered not to show LOH at this marker. Tumour (**C**) was homozygous at this marker and was noninformative.

A PCR-based method was used to detect loss of heterozygosity (LOH) at loci within, or close to, *ATM*, *BRCA1* or *TP53*. The allelic status of these genes was determined using six microsatellite markers, two for each gene: *D11S1816* (11q22–11q23, close to *ATM*), *D11S2179* (intron 34 of *ATM*), *D17S1322* (intron 19 of *BRCA1*), *D17S1323* (intron 12 of *BRCA1*), *TP53* (intron 1 of *TP53*) and *D17S786* (17p13, close to *TP53*). The sequences of the primers were: *D11S1816*, forward 5′-ATTGTGAAGCTAGGTGCTGGTG- 3′ and reverse 5′-AAAGAGATAAAACAGATTCTGGATG-3′; *D11S2179*, forward 5′-TAGGCAATACAGCAAGACCCTG-3′ and reverse 5′-GCACTGGAATACGATTCTAGCAC-3′; *D17S1322*, forward 5′-CTAGCCTGGGCAACAAACGA-3′ and reverse 5′-GCAGG- AAGCAGGAATGGAAC-3′; *D17S1323*, forward 5′-TAGGAGATGGATTATTGGTG-3′ and reverse 5′-AAGCAACTTTGCAATGAGTG-3′; *TP53*, forward 5′-CTTGTAGTCCTAGCTACTCAGCA-3′ and reverse 5′-CAAAACATCCCCTACCAAAC-3′; and *D17S786*, forward 5′-TACAGGGATAGGTAGCCGAG-3′ and reverse 5′-GGATTTGGGCTCTTTTGTAA-3′. The PCR amplification was carried out using 100 ng of DNA from tumour tissue or peripheral blood, 0.4 U of *Taq* polymerase, 0.2 mM deoxynucleotides and 2.5 mM MgCl_2_ in a total reaction volume of 10 *μ*l. The PCR conditions were 95°C for 12 min to activate *Taq* polymerase, followed by 40 cycles of denaturation (95°C, 45 s), annealing (55°C, 30 s) and extension (72°C, 45 s), the final elongation being performed at 72°C for 10 min. PCRs were run in a GeneAmp PCR 9600 thermocycler (PE Biosystems, Foster City, CA, USA). PCR amplifications omitting template DNA were included in each experiment as a control for contaminating DNA, and a housekeeping gene (*β*-actin) was used as an endogenous control. PCR products were electrophoresed on a 377 ABI PRISM sequencer, and the fluorescent signals from the different-sized alleles recorded and analysed using GENOTYPER (version 2.1) and GENESCAN (version 3.1). For an informative marker (heterozygous for the two alleles in blood specimens), the locus was considered to display LOH when there was a four-fold or greater difference in the relative allele intensity ratio between the tumour DNA and normal DNA (Figure 1

). Deletion of one allele of *ATM, BRCA1* or *TP53* was defined by either of the two markers showing LOH.

### Immunohistochemistry

Tissue specimens were fixed in 4% neutral-buffered formaldehyde, embedded in paraffin and cut into 4 *μ*m thick sections, which were deparaffinised for 3 × 5 min in xylene, rehydrated in graded alcohol and rinsed in Tris-buffered saline containing 0.1% Tween 20 (TBST). To improve antigen retrieval, dewaxed sections were immersed in 0.001 M EDTA, pH 8.0, heated for 30 min in a pressure cooker (TAC-10KS, Tatung, Taiwan), cooled to room temperature for 15 min, then rinsed briefly in TBST. Endogenous peroxidase was blocked by incubation for 5 min at room temperature in 3.5% hydrogen peroxide in TBST, followed by a TBST wash for 5 min. Nonspecific binding of antibodies was blocked by incubation of the sections for 30 min at room temperature with normal rabbit serum (1 : 5 in TBST). After washes with TBST, primary mouse monoclonal antibodies were added and the section incubated in a moist chamber for 1 h at room temperature. Following several rinses, biotinylated secondary antibody was applied for 30 min at room temperature, then the sections were rinsed and peroxidase-linked streptavidin (Dako, Copenhagen, Denmark) was added for 20 min. The chromogen was developed with AEC (Dako, Copenhagen, Denmark) and the slides were lightly counterstained with haematoxylin to provide cellular detail. The primary monoclonal antibodies used were specific for ATM (undiluted, ATX 08, NeoMarks, CA, USA), BRCA1 (1 : 125 dilution, SG11, Zymed, CA, USA) or TP53 (1 : 50 dilution, DO-7, DAKO, Copenhagen, Denmark). In negative controls, the primary antibody was replaced with TBST. In addition, the staining of infiltrating lymphocytes, stromal cells or adjacent normal epithelial cells within the tumours served as a normal control. Three pathologists independently evaluated the results semiquantitatively, and any discrepancy was resolved by joint review. Using previously reported criteria (Yoshikawa *et al*, 1999; Angèle *et al*, 2000), we compared the tumours to the adjacent normal epithelium (in which most cells show positive staining for ATM and BRCA1) and classified those tumours with more than 25% of ATM- or BRCA1-negative tumour cells as showing reduced expression of these proteins, all others being classed as showing normal expression. Since mutant TP53 proteins generally have a longer half-life than wild-type TP53 protein, which leads to their nuclear accumulation (Finlay *et al*, 1988), tumours with more than 20% of nuclear TP53-positive tumour cells were classed as showing aberrant expression, all others being classed as showing normal expression. For simplicity, both the above cases (‘reduced’ expression of ATM and BRCA1 and ‘aberrant’ expression of TP53) are referred to as ‘abnormal’ expression in the main text.

### Data analysis

The Mantel-extension *χ*^2^ test was used to examine the association between tumour grade and individual defective genes. Furthermore, to simultaneously examine all possible interactions between, and joint effects of, *ATM*, *BRCA1* and *TP53*, multigenetic analysis based on logistic regression was performed. All statistical analyses were performed using SAS version 8.0 software (SAS Institute Inc., USA).

## RESULTS

### Genetic deletion and tumour grade

**Figure 2:**
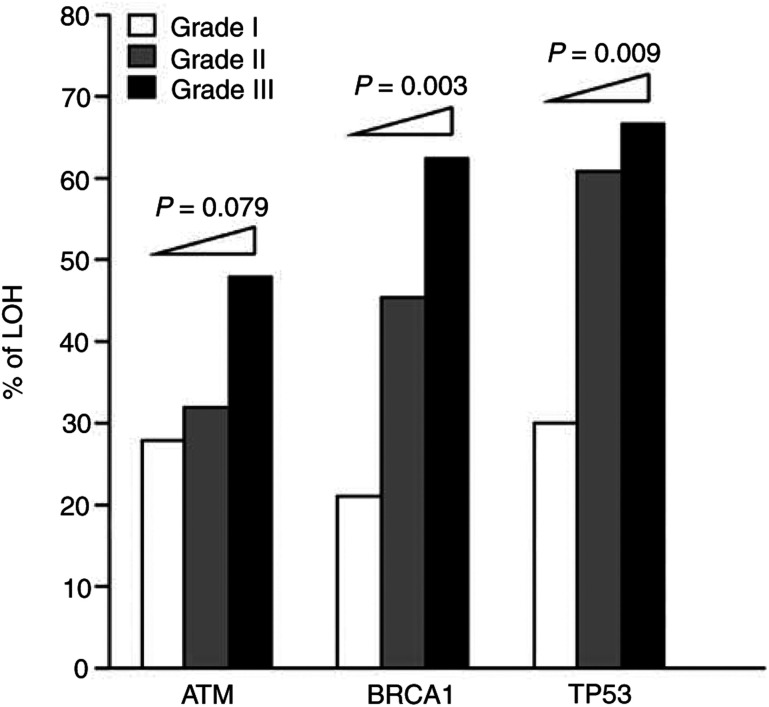
Frequency of LOH at the *ATM, BRCA1* or *TP53* locus in early-onset (**⩽**35 years of age) infiltrating ductal carcinoma of the breast stratified by pathological grade. The *P-*values of increasing trend were estimated using the Mantel-extension *χ*^2^ test (*N*=56).

Using four intragenetic markers (*D11S2179* for *ATM*, *D17S1322* and *D17S1323* for *BRCA1* and *TP53* for *TP53*) and two adjacent markers (*D11S1816* for *ATM* and *D17S786* for *TP53*), our results showed that, after excluding noninformative tumours (six out of 74 at *ATM*, nine out of 74 at *BRCA1* and 10/74 at *TP53*), the overall frequencies of LOH were 36. 8% for *ATM*, 44.6% for *BRCA1* and 53.1% for *TP53* in these early-onset breast tumours. These frequencies are similar to those reported previously (Shen *et al*, 2000). As shown in Figure 2

, even in grade I tumours, the LOH frequencies were 27.8% for *ATM*, 21.1% for *BRCA1* and 30.0% for *TP53*. When an association between the frequency of LOH at individual genes and tumour grade (tumour differentiation) was examined using the Mantel-extension *χ*^2^ test for trends, *BRCA1* and *TP53* showed a trend to a significant increase in LOH frequency in more poorly differentiated tumours (*P*=0.003, *P*=0.009, respectively).

### Abnormal protein expression and tumour grade

**Figure 3:**
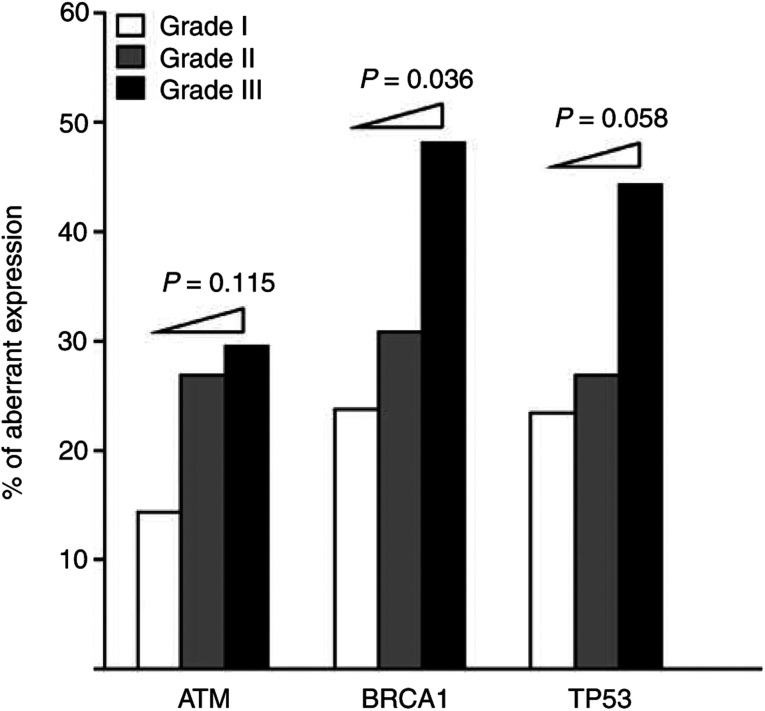
Frequency of abnormal expression of ATM, BRCA1 and TP53 in early-onset (⩽35 years of age) infiltrating ductal carcinoma of the breast stratified by pathological grade. The *P-*values were estimated using the Mantel-extension *χ*^2^ test (*N*=74).

Immunohistochemical staining showed that the frequency of abnormal expression was 24.3% for ATM, 32.1% for BRCA1 and 28.4% for TP53, and that high-grade tumours tend to have an increased frequency of abnormal expression of these three proteins (Figure 3

). When the frequency of abnormal expression of individual proteins was correlated with tumour grade using the Mantel-extension *χ*^2^ test for trends, there was a significant trend to an increase in abnormal BRCA1 expression with higher grade (*P=*0.036) (Figure 3).

### Coexistence of LOH and abnormal expression in the same gene

We then examined whether a combination of LOH and abnormal protein expression in the same gene was associated with poor differentiation. The results showed that, for *ATM*, combined LOH and abnormal expression was not associated with tumour grade (Table 1

**Table 1:** Genetic deletion (detected as loss of heterozygosity (LOH)) and abnormal expression (detected by immunohistochemical staining) of the DNA double-strand-break checkpoint/repair genes, *ATM*, *BRCA1* and *TP53*, as a function of pathological grade in breast cancer

		**Pathological grade**			
**Inactivating effects**	**No. of informative tumours**	**I**	**II**	**III**	***P*-value^a^ for trend**
*ATM*	68				NS
No LOH plus normal expression	33	13 (72.2%)	12 (48.0%)	8 (32.0%)	
Either LOH or reduced expression	30	3 (16.7%)	11 (44.0%)	16 (64.0%)	
LOH plus reduced expression	5	2 (11.1%)	2 (8.0%)	1 (4.0%)	
					
*BRCA1*	65				0.0003
No LOH plus normal expression	22	12 (63.2%)	7 (31.8%)	3 (12.5%)	
Either LOH or reduced expression	33	6 (31.6%)	13 (59.1%)	14 (58.3%)	
LOH plus reduced expression	10	1 (5.2%)	2 (9.1%)	7 (29.2%)	
					
*TP53*	64				0.0161
No LOH plus normal expression	23	11 (55.0%)	7 (30.4%)	5 (23.8%)	
Either LOH or aberrant expression	29	7 (35.0%)	13 (56.5%)	9 (42.9%)	
LOH plus aberrant expression	12	2 (10.0%)	3 (13.1%)	7 (33.3%)	

a *P-*values estimated using the Mantel-extension χ^2^chi-squared test.

). However, although the number of cases showing both LOH and abnormal expression in the same gene in each pathological grade was small, for *BRCA1* and, to a lesser extent, *TP53*, these combined abnormalities were frequently seen in association with a high tumour grade (*P*=0.0003, *P*=0.016, respectively). These results are consistent with the idea that several important DSB checkpoint/repair genes could be inactivated by different mechanisms, and that tumours with LOH and/or abnormal expression in the same gene are more likely to exhibit poorer differentiation.

### Defective *BRCA1* is the most significant indicator, but defective *ATM* or *TP53* provide an additional effect

The demonstration of a significant association between tumour grade and abnormality, either LOH or abnormal expression, at each DSB checkpoint/repair gene prompted us to ask which gene played the most critical role. Logistic regression analysis was performed to resolve this issue statistically. Since the age at the time of tumour onset is an important determinant for tumour grade (Jones and Laird, 1999; Bogdani *et al*, 2002), we included the patients' ages in this multivariate model. The analysis showed that LOH at *BRCA1* (*P*=0.02) and abnormal expression of BRCA1 protein (*P*=0.03) were the only two factors significantly and independently associated with poor pathological grade (Table 2

**Table 2:** Logistic regression and odds ratio showing the relationship between pathological grade and either LOH or abnormal expression of *ATM*, *BRCA1* or *TP53*

**Variable**	***β* regression coefficient**	**OR (95% CI)^a^**	***P-*value**
LOH at *ATM*	−0.08	0.93 (0.29–2.96)	NS
LOH at *BRCA1*	1.48	4.38 (1.25–15.41)	0.02
LOH at *TP53*	0.61	1.84 (0.59–5.71)	NS
Reduced ATM expression	−0.13	0.88 (0.19–4.04)	NS
Reduced BRCA1 expression	1.35	3.84 (1.13–13.05)	0.03
Aberrant TP53 accumulation	0.55	1.74 (0.46–6.57)	NS

a The odds ratio was adjusted for age (⩽30 years *vs* >30 years).

). This identification of the unique importance of *BRCA1*, however, did not exclude a joint effect of *BRCA1* and other functionally related genes, and we therefore examined whether abnormalities in *ATM* or *TP53* conferred an additional risk in cases in which *BRCA1* was abnormal. The result of logistic regression showed a more significant association with high grade in tumours with abnormalities in *BRCA1* and *ATM* and/or *TP53* (*P*=0.001) than in tumours with *BRCA1* abnormalities alone (*P*=0.09) (Table 3

**Table 3:** Logistic regression analysis of breast tumour pathological grade as a function of abnormalities in *BRCA1* alone or abnormalities in *BRCA1* plus abnormalities in *ATM* and/or *TP53*

**Genetic deletion and/or protein abnormalities**	***β* regression coefficient**	***P-*value^a^,^b^**
*BRCA1* alone^c^	2.43	0.090
*BRCA1* combined with *ATM* and/or *TP53*^d^	3.90	0.001

a The test was adjusted for age (⩽30 years *vs* >30 years).

b The reference group was defined as tumours with no LOH at, and no abnormal expression of, all three genes (*ATM*, *BRCA1* and *TP53*).

c Abnormalities in *BRCA1* (LOH and/or abnormal expression) alone.

d Abnormalities in *BRCA1* combined with abnormalities in *ATM* and/or *TP53* (either LOH or aberrant expression).

).

### Multiple defective genes are seen in high-grade tumours

Support for our hypothesis that abnormalities in individual genes in the DSB checkpoint/repair pathway might act in combination came from the observation of a significant association between poor tumour differentiation and an increased number of these genes showing LOH. As shown in Table 4

**Table 4:** Pathological grade as a function of the number of defective double-strand-break (DSB) checkpoint/repair genes harboured by individual tumours, detected as either loss of heterozygocity (LOH) or abnormal expression

	**Pathological grade**			
	**I**	**II**	**III**	
	**No. (%)**	**No. (%)**	**No. (%)**	***P-*value^a^**
No. of genes (*ATM*, *BRCA1* or *TP53*) with LOH (*N*=56)^b^				0.002
None	10 (62.5%)	4 (20.0%)	1 (5.0%)	
One gene	2 (12.5%)	5 (25.0%)	7 (35.0%)	
Two or three genes	4 (25.0%)	11 (55.0%)	12 (60.0%)	
				
No. of proteins (ATM, BRCA1 or TP53) showing abnormal expression (*N*=74)				0.026
None	12 (40.0%)	12 (40.0%)	6 (20.0%)	
One protein	5 (21.7%)	7 (30.4%)	11 (47.8%)	
Two or three proteins	4 (19.1%)	7 (33.3%)	10 (47.6%)	

a Mantel-extension test for trend comparing the frequency distribution of pathological grades in tumours showing LOH at (abnormal expression of) one or two/three DSB checkpoint/repair genes (*ATM*, *BRCA1* and *TP53*) and in tumours with no LOH at (no abnormal expression of) any of these three genes.

b The 56 tumours were informative (heterozygous for the two alleles of either of the two microsatellite markers reflecting the genetic status of the individual genes) at *ATM, BRCA1* and *TP53*.

, 62% of grade I tumours showed no evidence of LOH at *ATM, BRCA1* or *TP53*, whereas all three genes were intact in only 5% of grade III tumours (*P*=0.002). Furthermore, the results of the Mantel-extension *χ*^2^ test for trends confirmed the finding that an increased number of genes showing abnormal expression correlated with poor tumour differentiation (*P*=0.026). However, since breast cancer always displays a high degree of intratumour heterogeneity in each individual tumour, it was possible that our finding of abnormal protein expression based on the overall abnormal frequency described above might fail to take into account intratumoral differences in protein expression. To exclude this possibility, we used serial sections to analyse the same region for the expression and subcellular location of the proteins of interest. Two typical examples are shown in Figure 4

**Figure 4:**
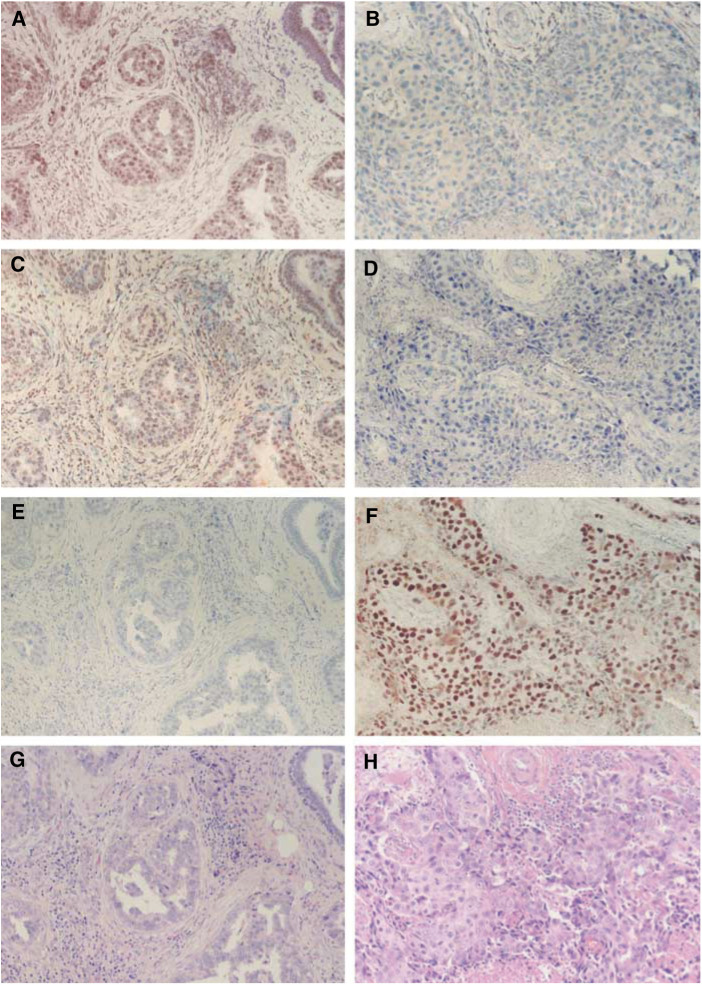
Expression of DNA DSB checkpoint/repair proteins in infiltrating ductal carcinoma of the breast. Adjacent serial sections from a grade I tumour (differentiation score of 5 points) (**A**, **C**, **E**, **G**) or a grade III tumour (differentiation score of 8 points) (**B**, **D**, **F**, **H**) were stained with haematoxylin–eosin (**G**, **H**) or with antibodies against ATM (**A**, **B**), BRCA1 (**C**, **D**) or TP53 (**E**, **F**). Original magnification × 200.

, in which a low-grade tumour displayed strong nuclear staining for ATM and BRCA1 and no TP53 accumulation, while a high-grade tumour displayed abnormal expression of all three proteins.

## DISCUSSION

The rationale for a mutator contributing to the genomic instability that leads to cancer progression is that, instead of being a single-gene disease, cancer is caused by aberrations arising from a complex interconnecting network of multiple regulatory genes involved in normal growth control processes and the maintenance of genomic stability (Orr-Weaver and Weinberg, 1998; Loeb and Loeb, 2000). This rationale supports our approach of simultaneously examining the role of three important genes (*ATM*, *BRCA1* and *TP53*) involved in the DSB checkpoint/repair pathway. Although abnormalities of individual DSB checkpoint/repair genes or proteins have been reported (Waha *et al*, 1998; Wilson *et al*, 1999; Angèle *et al*, 2000), almost all of these have been studied in isolation without considering the common pathway involved. In contrast, in the present study, using a largely homogeneous group of 74 early-onset IDCs and a multigenetic model, we have evaluated the tumorigenic contribution of LOH and/or abnormal expression of these genes. In terms of methodology, we selected intragenic markers and used laser capture microdissection for our LOH analysis to provide an unbiased estimate of the genetic status of these genes in cancer cells, and our careful examination of protein expression based on serial sections excluded the possibility of an effect of intratumour heterogeneity. The results (Figure 2) showed that loci harbouring *ATM*, *BRCA1* or *TP53* displayed a high frequency of LOH in low-grade tumours, suggesting that genetic deletion of these DSB checkpoint/repair genes frequently occurs during the early stage of breast tumour. This is consistent with the results of our previous study (Shen *et al*, 2000), which suggested that breast cancer progression is driven by DSB-initiated chromosomal instability. In addition, our immunohistochemical study (Figure 3) showed that high-grade tumours tended to have an increased frequency of abnormal expression of these three proteins and, in particular, that abnormal BRCA1 expression was related to poor differentiation (Figure 3), supporting an important contribution of DSB checkpoint/repair genes to breast cancer pathogenesis.

The present study also examined whether combined LOH and abnormal expression occurring at the same gene could contribute to the pathogenesis of breast cancer, and our findings for *TP53* and *BRCA1* seem to be in line with this hypothesis (Table 1). However, different mechanisms are involved in the effects of combined LOH and abnormal expression of *TP53* and *BRCA1*. In sporadic cancers, LOH at the *TP53* locus is usually accompanied by somatic mutation at *TP53* (Yang *et al*, 1997; Kurose *et al*, 2002), leading to abnormal accumulation of this protein. Thus, a high frequency of poorly differentiated tumours showing both LOH at, and aberrant expression of, *TP53* can be explained mechanistically. In contrast, *BRCA1* mutations are rare in sporadic breast cancer, suggesting that *BRCA1* is inactivated by nonmutational mechanisms (Papa *et al*, 1998), since epigenetic mechanisms (e.g. promoter hypermethylation), manifested as abnormal expression, have been shown to be involved in abrogating the function of certain tumour suppressor genes (such as *BRCA1*), which are already targeted by LOH (Dobrovic and Simpfendorfer, 1997; Jones and Baylin, 2002). Our finding that combined LOH and abnormal expression in *BRCA1* was associated with a higher tumour grade therefore re-emphasises the importance of the different mechanisms that may affect expression, and this finding is of particular tumorigenic importance in sporadic cancer, as the frequency of somatic mutation in many tumour suppressor genes involved in regulating genomic stability is extremely low. In the present study, we also found that LOH was more frequent than abnormal protein expression at *ATM*, *BRCA1* and *TP53*. One possible explanation for this is that expression of the remaining allele might increase to compensate for the loss of expression due to the deleted allele (Liao *et al*, 2003).

The observation that the only gene of the three tested found by multigenetic logistic regression analysis to be independently associated with tumour differentiation was *BRCA1* (Table 2) is consistent with the tumour spectrum observed in familial cancer syndromes caused by mutation of *ATM*, *BRCA1* or *TP53*, with only *BRCA1* being specifically associated with familial breast cancer (Holt *et al*, 1996). However, the present study showed that a joint effect of abnormalities, either LOH or abnormal expression, in *BRCA1*, *ATM* and *TP53* is also an important factor associated with poor differentiation (Tables 3 and 4). This effect can be explained by known mechanisms involving interactions between BRCA1, ATM and TP53 (Xu *et al*, 1999; Gatei *et al*, 2000; Wang, 2000; Khanna and Jackson, 2001), including (i) ATM serves as the upstream sensor and, upon DSB formation, phosphorylates BRCA1 and TP53 to trigger DSB repair and cell cycle regulation and (ii) for breast cancer formation, breast epithelium cells that are genomically unstable because of defective BRCA1-associated repair also need to undergo checkpoint inactivation (such as ATM or TP53) in order to escape checkpoint surveillance. More importantly, our demonstration that defective DSB checkpoint/repair genes in a common functional pathway may act together, leading to poor differentiation of breast cancer, is consistent with recent evidence suggesting the joint contribution of different genes in disease aetiology and cancer development. The combination of heterozygous abnormalities in different, but functionally related, genes is known to play a causal role in the pathogenesis of certain genetic syndromes (Cressman *et al*, 1999; Balmain, 2002). Furthermore, support for a joint carcinogenic effect comes from previous observational studies. For instance, there is a trend towards an increased risk of breast cancer in women harbouring a greater number of putative high-risk genotypes of oestrogen-metabolising genes or nonhomologous end-joining genes (Huang *et al*, 1999; Fu *et al*, 2003). Our findings provide additional support for the possibility of a joint effect of defects in different genes and emphasise the need to examine the whole tumorigenic pathway to obtain a better insight into the molecular changes involved in cancer development and progression.
